# Outcomes of Cardiac Contractility Modulation: A Systematic Review and Meta-Analysis of Randomized Clinical Trials

**DOI:** 10.1155/2019/9769724

**Published:** 2019-06-17

**Authors:** Ramy Mando, Akshay Goel, Fuad Habash, Marwan Saad, Karam Ayoub, Srikanth Vallurupalli, Waddah Maskoun

**Affiliations:** ^1^Department of Internal Medicine, Beaumont Health System, Royal Oak, MI, USA; ^2^Department of Cardiovascular Medicine, University of Arkansas for Medical Sciences, USA; ^3^Department of Cardiovascular Medicine, University of Kentucky, Lexington, KY, USA; ^4^Department of Cardiovascular Medicine, Henry Ford Hospital, Detroit, MI, USA

## Abstract

**Background:**

Cardiac contractility modulation (CCM) is a device therapy for systolic heart failure (HF) in patients with narrow QRS. We aimed to perform an updated meta-analysis of the randomized clinical trials (RCTs) to assess the efficacy and safety of CCM therapy.

**Methods:**

We conducted a systematic review and meta-analysis of randomized clinical trials (RCTs) between January 2001 and June 2018. Outcomes of interest were peak oxygen consumption (peak VO2), 6-Minute Walk Distance (6MWD), Minnesota Living with Heart Failure Questionnaire (MLHFQ), HF hospitalizations, cardiac arrhythmias, pacemaker/ICD malfunctioning, all-cause hospitalizations, and mortality. Data were expressed as standardized mean difference (SMD) or odds ratio (OR).

**Results:**

Four RCTs including 801 patients (CCM n = 394) were available for analysis. The mean age was 59.63 ± 0.84 years, mean ejection fraction was 29.14 ± 1.22%, and mean QRS duration was 106.23 ± 1.65 msec. Mean follow-up duration was six months. CCM was associated with improved MLWHFQ (SMD -0.69, p = 0.0008). There were no differences in HF hospitalizations (OR 0.76, p = 0.12), 6MWD (SMD 0.67, p = 0.10), arrhythmias (OR 1.40, p = 0.14), pacemaker/ICD malfunction/sensing defect (OR 2.23, p = 0.06), all-cause hospitalizations (OR 0.73, p = 0.33), or all-cause mortality (OR 1.04, p = 0.92) between the CCM and non-CCM groups.

**Conclusions:**

Short-term treatment with CCM may improve MLFHQ without significant difference in 6MWD, arrhythmic events, HF hospitalizations, all-cause hospitalizations, and all-cause mortality. There is a trend towards increased pacemaker/ICD device malfunction. Larger RCTs might be needed to determine if the CCM therapy will be beneficial with longer follow-up.

## 1. Introduction

Current treatment options for patients with systolic heart failure (HF) target improving survival, quality of life, left ventricular (LV) function, and reducing HF-related hospitalizations. Therapy typically includes optimization of medical management, revascularization, managing valvular heart disease, and device therapy when appropriate (defibrillator and biventricular pacing).

Several studies have shown that cardiac resynchronization therapy (CRT) helps patients with wide QRS on 12 Lead ECG (>120 msec), an ejection fraction (EF) ≤ 35%, and New York Heart Association (NYHA) class II-IV symptoms [1–3]. Approximately 30% of those who meet the aforementioned criteria do not experience improvement with CRT. Moreover, about 50% of individuals with advanced HF do not meet criteria and are therefore not candidates for CRT [2, 4–6]. This creates a cohort of patients including those with advanced HF and a narrow QRS complex (< 120 msec) and nonresponders to CRT requiring new therapies to improve their overall quality of life.

Cardiac contractility modulation (CCM) is a modality that delivers a high voltage impulse to the right ventricular septum 30-40 msec after activation of cardiomyocytes during the absolute refractory period. In theory, this improves calcium handling and increases ventricular contractility with resultant improvement in exercise tolerance and functional capacity [7–9]. It is not clear if CCM may play a role in patients who are not good candidates for CRT devices (narrow QRS), nonresponders to CRT or in conjunction with CRT device therapy [10, 11]. Previous studies of CCM were small and likely underpowered to detect significant differences [12, 13]. With the publication of the recent FIX-HF-5C study, we performed a meta-analysis of the randomized clinical trials (RCTs) to assess the efficacy and safety of CCM therapy in patients with systolic HF and narrow QRS complex.

## 2. Methods

### 2.1. Search Strategy, Study Selection, and Data Extraction

A systematic review of PubMed, MEDLINE, and Cochrane Central Register of Controlled Trials was preformed from 2001 until June 2018 without any language restriction, according to the Preferred Reporting Items for Systematic Reviews and Meta-Analyses (PRISMA) guidelines [14]. We used the keywords “cardiac contractility modulation”, “heart failure”, and “systolic heart failure”. After eligible studies were retrieved, we screened their bibliographies for any potential missed studies through the initial search. Furthermore, prior meta-analyses were screened to ensure the inclusion of all eligible studies. Studies available for inclusion were (1) RCTs assessing safety, efficacy and outcomes of cardiac contractility modulation, (2) adult patients (≥18 years), (3) intervention group assigned to CCM, and (4) control group assigned to optimal medical therapy (OMT). Two independent authors extracted data on study characteristics, patient demographics, and quality assessments. Extracted data were revised by a third author to ensure accuracy. Discrepancies were resolved by consensus among authors. Study level data were extracted since individual subject data were not available.

### 2.2. Outcome Measures

Primary outcomes were (1) all-cause mortality, (2) all-cause hospitalizations, (3) worsening heart failure/hospitalizations, (4) incidence of cardiac arrhythmias defined as symptomatic supraventricular or ventricular arrhythmia and/or requiring intervention, and (5) pacemaker/ICD malfunctioning/missensing. Other heart failure outcomes of interest were (6) peak oxygen consumption (peak VO_2_, mL/kg/min) assessed by cardiopulmonary exercise testing, an important objective prognostic measure of peak aerobic capacity [15–17]; this is important to help differentiate true results from placebo effect, particularly in nonsham-controlled studies; (7) six-minute walk distance (6MWD) which is the distance covered, in meters, over 6 minutes of maximal self-paced walking [18]; (8) Minnesota Living with Heart Failure Questionnaire (MLHFQ) used to assess patient's perception of the consequences of HF on the physical, socioeconomic, and psychological aspects of life [19].

### 2.3. Assessment of Quality and Bias

The quality of the included trials and the risk of the bias were assessed by two independent reviewers using the components recommended by Cochrane Collaboration, including random sequence generation, allocation concealment, blinding of participants and personnel, blinding of outcome assessment, incomplete outcome data, selective reporting, and other sources of bias. Trials were considered low risk for bias if having < 2 high-risk components and high potential for bias if having > 4 high-risk components. The overall quality of evidence for each outcome was further assessed using GRADE (Grades of Recommendation, Assessment, Development and Evaluation) tool recommended by the Cochrane Handbook for Systematic Reviews of Intervention.

### 2.4. Statistical Analysis

Descriptive analyses were performed using weighted means and standard deviations (SD) for continuous variables and weighted frequencies for categorical variables. Random effect DerSimonian-Laird model was used to calculate standardized mean difference (SMD) for the continuous variable outcomes and odds ratio (OR) to estimate the effect sizes for other outcomes. Heterogeneity between the studies of each outcome was evaluated as well.

The sample size of each study was used as its weight. P-values (2-tailed) were considered statistically significant if less than 0.05. We calculated the confidence intervals (CIs) at the 95% level for the overall estimates effect. All statistical analyses were conducted using Revman 5.3. The Nordic Cochrane Centre, Copenhagen, Denmark, was used to conduct meta-analysis for outcome measures [22].

## 3. Results

### 3.1. Identified Studies

**Figure 1:**
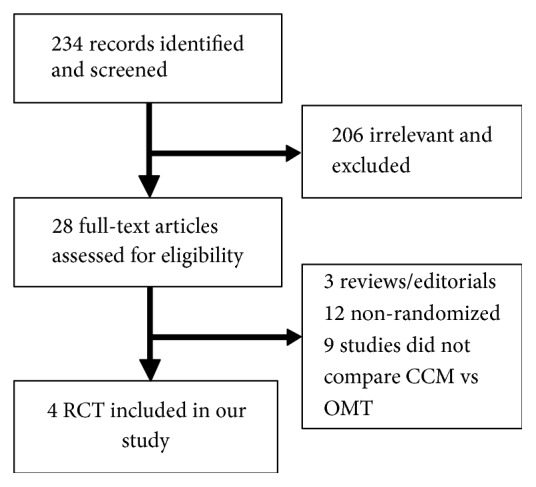
*A flow diagram of the search strategy conducted.* A flow diagram of the search strategy conducted. The purpose of this figure is to provide a graphical representation of the manner in which we conducted our search for RCT for CCM. PRISMA guidelines were followed.

Four RCTs including a total of 801 patients (394 patients with CCM) met our eligibility criteria [12, 13, 20, 21] (Figure 1). Mean age was 59.63 ± 0.84 years, 75.28% were males, and 63.67% had ischemic cardiomyopathy with mean ejection fraction 29.14 ± 1.22% and mean QRS duration of 106.23 ± 1.65 msec. Details about the trials' characteristics and patients' baseline demographics are summarized in Table 1. Baseline characteristics in these studies were comparable between CCM and non-CCM groups. A quality assessment and publication bias analysis was completed and can be found in the supplemental material (available here).

### 3.2. Outcome Measures

#### 3.2.1. Adverse Events and Total Mortality

**Figure 2:**
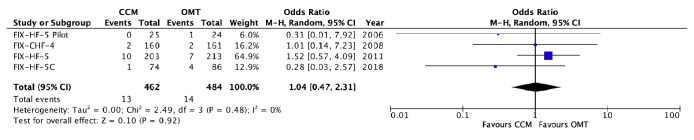
*Forest plot of all-cause mortality (postrandomization/device implantation).* A forest plot of the data available to us from the 4 RCTs assessing all-cause mortality in those with CCM compared to those with OMT alone. There was a nonsignificant difference in the rate of total hospitalizations between the two groups.

**Figure 3:**
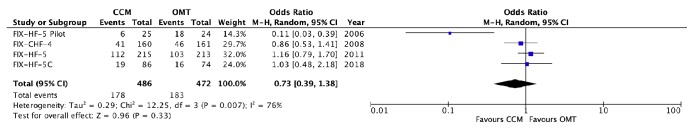
*Forest plot of total hospitalizations in the CCM groups versus the control groups.* A forest plot of the data available to us from the 4 RCTs assessing total hospitalizations in those with CCM compared to those with OMT alone. There was a nonsignificant difference in the rate of total hospitalizations between the two groups.

**Figure 4:**
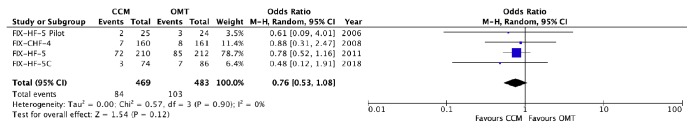
*Forest plot of worsening HF/HF-related hospitalizations in the CCM groups versus the control groups.* A forest plot of the data available to us from the 4 RCTs assessing worsening HF and HF-related hospitalizations in those with CCM compared to those with OMT alone. There was a nonsignificant trend in reduced HF hospitalizations in those with CCM.

**Figure 5:**
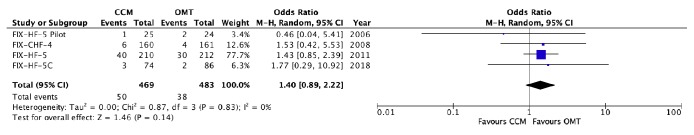
*Forest plot of cardiac arrhythmias in CCM groups versus the control groups.* A forest plot of the data available to us from the 4 RCTs assessing arrhythmias in those with CCM compared to those with OMT alone. There was a nonsignificant trend in reduced arrhythmic events favoring the OMT group.

**Figure 6:**
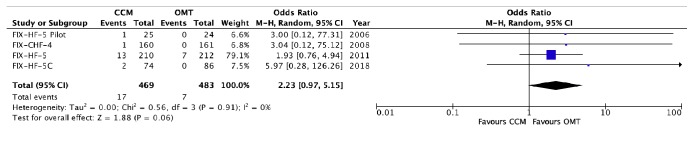
*Forest plot of pacemaker/ICD sensing defects/malfunction in CCM groups versus the control groups.* A forest plot of the data available to us from the 4 RCTs assessing pacemaker/ICD sensing defects/malfunction in those with CCM compared to those with OMT alone. There was a nonsignificant trend in increased sensing defect and malfunction in the CCM group.

The adverse events of each trial are detailed in Table 2. There was no significant difference between those receiving CCM + OMT intervention when compared to those receiving OMT alone with regard to worsening HF/HF hospitalizations (OR 0.76, 95% CI 0.53 – 1.08, p = 0.12, Figure 4), arrhythmic events (OR 1.40, 95% CI 0.89 to 2.22, p = 0.14, Figure 5), pacemaker/ICD malfunction/sensing defect (OR 2.23, 95% CI 0.97 to 5.15, p = 0.06, Figure 6), total hospitalizations (OR 0.73, 95% CI 0.39 to 1.38, p = 0.33, Figure 3), and total mortality (OR 1.04, 95% CI 0.47 to 2.31, p = 0.92, Figure 2). We were unable to assess difference in cardiac vs noncardiac mortality as cause of death was not specified for a majority of patients. Furthermore, it was not clear whether all the arrhythmias reported were symptomatic or required any particular intervention.

#### 3.2.2. Peak *VO*_2_

Evaluating the peak VO_2_ response was limited by the variation in data reporting among the four randomized controlled trials and we were not able to perform analysis on the pooled data due to that. Overall, the mean difference of peak VO_2_ favored the CCM group in all studies. The FIX-HF-5 pilot study reported an overall mean difference of 0.2 mL O_2_/kg/min favoring the treatment group, although this was not statistically significant [12]. The FIX-CHF-4 study also reported positive findings of 0.52 mL O_2_/kg/min in the treatment group relative the sham therapy. [13]. The FIX-HF-5 study by Kadish et al. reported improvement of 0.65 mL O_2_/kg/min [20]. Lastly, the most recent FIX-HF-5C study demonstrated improvement in VO_2_ of 0.84 mL O_2_/kg/min [21]. All of the latter trials reached statistical significance.

#### 3.2.3. Six-Minute Walking Distance

**Figure 7:**
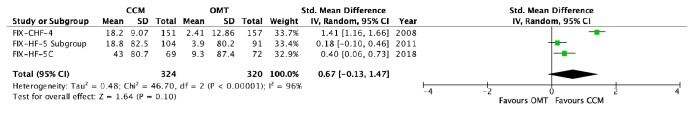
*Forest plot of the six-minute walking distance in the CCM groups versus the control groups.* A forest plot of the data available to us from the 4 RCTs assessing 6MWD in those with CCM compared to those with OMT alone. There was no statistically significant difference in 6MWD between these two groups.

There was no significant difference in the 6MWD in the CCM group compared to those receiving OMT alone (SMD 0.67-meters, 95% CI -0.13 to 1.47, p = 0.10, Figure 7). In the FIX-HF-5 pilot study there was a nonsignificant increase in the 6MWD in the CCM group at 24 weeks [12]. Individual data from this study was not available for inclusion in our analysis. In the FIX-HF-5, it is important to note that, for all comers, they found a nonsignificant improvement in the 6MWD in the CCM group (~ 10 m). The only data available for inclusion into our analysis was that of the “responders” subgroup (LVEF ≥ 25% and ≤ 45%) of FIX-HF-5 and FIX-HF-5C, as shown in Figure 2 [20, 21].

#### 3.2.4. Quality of Life Measured by MLHFQ

**Figure 8:**
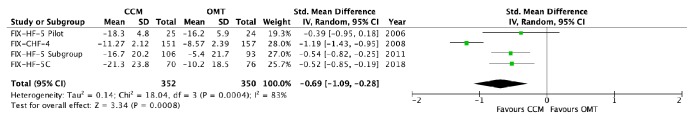
*Forest plot of the MLWHFQ in the CCM groups versus the control groups.* A forest plot of the data available to us from the 4 RCTs assessing MLWHFQ in those with CCM compared to those with OMT alone. There was a statistically significant difference between these two groups favoring CCM.

Significant improvement in quality of life in those receiving CCM intervention was noted based on the decrease in MLWHFQ score (SMD -0.69, 95% CI -1.09 to -0.28, p < 0.01, Figure 8). In the FIX-HF-5 study CCM therapy improved the MLWHFQ for the total cohort, and again only the same subgroup was available for our statistical analysis [20, 21]

## 4. Discussion

Several studies suggest cardiac contractility modulation might be a promising therapy for systolic heart failure patients with narrow QRS who are already on OMT. CCM therapy enhances LV contractility independent of myocardial synchrony and QRS duration by delivering signals 30-40 msec after activation of myocytes during the absolute refractory period [20, 23–25]. These signals are nonexcitatory and therefore should not initiate contraction or modify myocyte activation [7, 12]. It is believed these signals help in regulating calcium cycling by cardiac myocytes through phosphorylation of proteins and expression of genes involved in this process. The modulation of calcium entry into myocytes during the refractory period is thought to lead to augmented contractility [7, 26, 27]. For these reasons, CCM may play an important role in managing patients in a subset of heart failure with reduced ejection fraction.

Our meta-analysis included four randomized clinical trials with a total of 801 enrolled patients comparing CCM therapy versus OMT. The objective outcome of peak VO_2_ was reported in different ways in the four studies; however, the mean difference of peak VO_2_ favored the CCM group in all studies. Overall, we found that CCM therapy improves QOL measured by MLWHFQ when compared to optimal medical management alone and trend towards improving the 6MWD (p = 0.10). In the most recent RCT by Abraham et al. peak VO_2_, MLWHFQ, NYHA functional class, and 6MWD were all better in the CCM treatment versus control group [21]. The study included a total of 160 patients with NYHA functional class III or IV symptoms, QRS duration <130 msec, and ejection fraction ≥25% and ≤45% patients. The improvements seen were less than achieved with previous CRT trials [2, 28, 29]. A recent meta-analysis for the individual patient's data of the three prior RCTs suggested modest beneficial role of CCM in improving exercise capacity and quality of life. [30]. This analysis included only three randomized controlled trials and did not address worsening heart failure/hospitalizations, incidence of cardiac arrhythmias, pacemaker/ICD malfunctioning/missensing, all-cause hospitalizations, or all-cause mortality. Certain groups experienced more robust improvements in 6MWD in that meta-analysis. These groups included those of male gender, those with ischemic cardiomyopathy, and those with an EF between 25-45% [30].

In the FIX-HF-5 pilot study, there was a nonsignificant improvement in the 6MWD between the intervention group and the control group [12]. Similarly, in the FIX-HF-5 study by Kadish et al. there was a 10-meter improvement in the 6MWD in the CCM group when compared to the control group which was not statistically significant [20]. It is important to point out that we were only able to include the “responders” subgroup of patients from Kadish et al. study in our 6MWD and MLWHFQ statistical analysis as this population was identified as better responders to CCM therapy [20, 21]. In our study, the improvements seen in the MLWHFQ may well be significant statistically given the more robust sample size included in this analysis. This is less likely to be clinically relevant given the minimal improvement and potential for placebo effects in some of the included studies. Similarly, the trend of 0.67-meter improvement noted in 6MWD is likely far from relevance in light of risks associated with device placement.

The FIX-HF-5 pilot study found no difference in QOL using the MLWHFQ due to similar improvements in the MLWHFQ between both groups (CCM on and CCM off). This raised the suspicion that the benefits of CCM may be related to placebo effect [22]. However, parameters such as pVO_2_ provide objective evidence of improved functional capacity overall improved in the 4 studies.

In our meta-analysis we found trend towards lower rates of worsening HF (p = 0.12); however, we also identified a trend towards increased arrhythmic events (p = 0.14). Total hospitalization and mortality were similar in the two treatment groups in our study. An earlier meta-analysis of 3 randomized studies concluded that CCM was not associated with a worse prognosis when compared to OMT [31]. The FIX-HF-5 pilot study showed significantly reduced all-cause hospitalizations for the CCM group when compared with OMT alone (84% versus 62%, respectively) despite the CCM population having worse baseline characteristics [12]. However, these results were not reproducible in the subsequent two trials which revealed no statistically significant difference in hospitalization rates alone [13, 20, 21]. The primary safety end point of the study, which was a noninferiority assessment of the composite of all-cause mortality and all-cause hospitalization, was satisfied in the FIX-HF-5 study [20]. In FIX-HF 5C, CCM led to significant improvement in the* combined* end points of survival free of cardiac death and heart failure hospitalizations [21].

Interestingly, in our study we found that sensing defects may be an issue in those receiving CCM despite the routine evaluation for oversensing in patients with ICDs during CCM implantation (p = 0.06) [9]. About 80% of the cohort in our meta-analysis already had implantable devices (ICD or pacemaker). Implantation of another device and lead system may predispose to an increase in complication rates (Table 2) such as leads dislodgment, pericardial effusion, DVT, lead fracture, perforation, pocket infection, and need for extraction. Device complications are important to ascertain as they may lead to increased hospitalizations and even mortality irrespective of the underlying CHF. The primary safety endpoint for FIX-HF-5C study was defined as the proportion of subjects who did not experience either an Optimizer device-related complication or a procedure-related complication by 24 weeks and was met in that study (89.7% complication-free rate) [21].

Importantly, all studies were limited by short follow-up periods which preclude the assessment of any long-term benefit. CCM may become the device therapy of choice in patients with systolic HF and narrow QRS based on symptomatic improvement and reducing HF admissions. However, the clinical benefits will be limited if no improvement in total mortality and/or total hospitalizations is achieved.

Several studies of longer follow-up assessing mortality following CCM placement have shown promising outcomes [32–36]. The longest follow-up among these studies was by Liu et at with 6 years of follow-up of 41 patients demonstrating significant improvement in mortality and heart failure hospitalization in the CCM group for patients with EF ≥ 25-40% [34]. Similar studies enrolling 81 patients followed for 3 years and another enrolling 68 patients followed for 5 eyars also demonstrated better than expected mortality rates [33, 36]. The first of these studies, a retrospective analysis of 54 consecutive patients receiving CCM with 3 years of follow-up, revealed an all-cause mortality similar to that predicted by the Seattle Heart Failure Model (SHFM) [32]. The most recent study included retrospective review of 140 patients in the CCM registry divided patients into those with an EF 25-45%, EF 24-34% and an EF 35-45%. This study revealed mortality similar to that predicted by the SHFM in all comers (i.e., EF 25-45%) and in those with an EF between 24-34%. They reported a significant improvement in 3-year mortality compared to expected in the 57 patients with an EF between 35 and 45% (88% vs 74.7%) [35]. A powered large randomized control trial with long-term follow-up which is needed to address such important outcomes will be different.

Adverse events and procedure complications are currently under evaluation by two ongoing studies: Continued Access Protocol for the Evaluation of the OPTIMIZER Smart System (FIX-HF-5CA) is evaluating the serious adverse device Events in 250 participants and Evaluation of the Safety and Efficacy of the 2-lead OPTIMIZER® Smart System (FIX-HF-5C2) is evaluating optimizer device- or procedure-related complication in 60 participants. The recently published study by Anker et al. revealed two severe ICD related adverse events and ten severe adverse events related to arrhythmias [35]. With the technological advancements, the potential to merge ICD or CRT and CCM devices is feasible and might reduce the risk of the procedure and the device-related complications.

## 5. Future Direction of CCM Therapy

We feel that the future direction of CCM is to conduct randomized controlled trials with long follow-up to evaluate heart failure and total hospitalizations and death. Furthermore, objective data such as the impact of CCM on left ventricular end systolic and diastolic diameter and ejection fraction need to be evaluated. Lastly, integration of CCM technology into the current implanted cardiac devices will hopefully significantly reduce the complications due to additional device and procedure.

## 6. Limitations

The meta-analysis is based on study level data since patient level data was largely unavailable. Variations in study design are noted in Table 1. Sources of bias within these studies include lack of blinding as well as lack of sham procedure in some studies. Patients with atrial arrhythmias and atrial fibrillation were excluded but current studies suggest that CCM could be feasible in those with permanent atrial fibrillation [10]. The short follow-up duration precludes accurate assessment of benefits in hard clinical outcomes.

## 7. Conclusion

Our meta-analysis demonstrates that short-term treatment with CCM improves QOL measured by MLFHQ. The clinical relevance of this improvement might be insignificant. Trend towards higher incidence of pacemaker/ICD device malfunction was noted in the CCM group when compared to those receiving optimal medical therapy alone. We found no difference with regard to 6MWD, arrhythmic events, HF hospitalizations, total hospitalizations, and mortality between the two groups. The Food and Drug Administration (FDA) recently issued memorandum addressing CCM as a breakthrough designated device that might help patient population with limited additional therapy options that can improve heart failure symptoms and hospital admission and survival. Due to the better outcome with longer follow-up and less severe LV dysfunction, larger randomized controlled trials with a longer follow-up periods are needed to determine whether CCM should be widely accepted or not as a therapeutic option for patients with advanced, narrow QRS complex systolic HF or for those who did not experience symptomatic improvement from CRT.

## Figures and Tables

**Table 1:** Studies characteristics and patient demographics.

	FIX-HF-5 Pilot [12]	FIX-CHF-4 [13] (Group 1/Group 2)	FIX-HF-5 [20]	FIX-HF-5C [21]
*Year *	2006	2008	2011	2018

*Sample Size (N) *	49	164	428	160

*Patients Withdrawn*	0	5 (group 1) 4 (group 2)	17 (OMT) 6 (CCM)	3 (OMT) 1 (CCM)

*Study Design*	CCM to all patients, 25 active CCM vs 24 inactive CCM	Crossover Study: CCM to all patients; Group 1 (N=80; CCM first 3 months), Group 2 (N=84; sham first 3 months)	CCM Implant (N = 215) vs OMT (N=213)	CCM Implant (N = 74) vs OMT (N = 86)

*Ejection Fraction Inclusion Criteria *	< 35%	< 35%	≤ 35%	≥25% and ≤ 45%

*Mean Follow up *	6 months	6 months	6 months (noninferiority at 12 months)	6 months

*Outcomes *	NYHA Class, 6MWD, Stress Test, Holter Monitoring	Holter Monitoring. Changes in peak VO_2_, MLHFQ, and 6MWD at the end of 12 and 24 weeks.	Ventilatory anaerobic threshold, peak O2, MLHFQ, non-inferiority based on mortality and hospitalization with 12.5% allowable delta (12 months)	Peak VO_2_, MLWHFQ, NYHA Class, 6MWD, safety assessed by percentages of patients free of device-related events.

*Study Centers *	Single Center – Lone Star Arrhythmia and Heart Failure Center - Texas	Single Center - Germany	50 US Centers	42 Centers (US, Germany, and Czechia).

*Mean age* (CCM/OMT)	52 ± 15.0 59.6 ± 12.0	58.9 ± 9.8 59.9 ± 10	58.09 ± 12.79 58.55 ± 12.33	63 ± 11 63 ± 11

*Male* (CCM/OMT)	68%/71%	88.8%/81%	73.5%/70.9%	73%/79.1%

*SBP (mmHg)* (CCM/OMT)	118.6 ± 19.7 115 ± 20.6	114.7 ± 17.0 117.1± 17.9	116.65 ± 19.48 115.61 ± 17.61	123 ± 18 126 ± 19

*QRS Duration (msec)* (CCM/OMT)	109.2 ± 15.8 101.3 ± 14.2	119.9 ± 28.3 116.3 ± 26.6	101.63 ± 15.30 101.51 ± 12.81	103 ± 13.0 103.6 ± 12.1

%* ICD/Pacemaker* (CCM/OMT)	88% (22) 83% (20)	68.3% (55) 59.4% (50)	96% (207/215) 95% (202/213)	87.8% (65/74) 84.9% (73/86)

*6MWD Baseline (m)* (CCM/OMT)	321 352 ± 95.4	386 ± 103 394 ± 102	326.38 ± 82.10 323.99 ± 92.44	317 ± 88 324 ± 90

*NYHA Class Included*	III and IV	II and III	III and IV	III and IV

*NYHA Class III* (CCM/OMT)	100% 96%	72.5% 80%	91.16% 85.92%	86.5% 90.7%

*Ischemic Cardiomyopathy* (CCM/OMT)	64% 67%	63.8% 56%	64.7% 66.7%	62.2% 59.3%

*Baseline LVEF (*%) (CCM/OMT)	24.9 ± 6.5 31.4 ± 7.4	29.3 ± 6.6 29.8 ± 7.8	25.74 ± 6.60 26.09 ± 6.54	33 ± 6 33 ± 5

*Ventilatory Anaerobic Threshold (VAT)* (CCM/OMT)	10.6 ± 2.4 12.3 ± 2.5	Not documented	10.95 ± 2.24 10.97 ± 2.18	Not documented

*Peak O2 Consumption* (CCM/OMT)	14.3 ± 2.8 16.0 ± 2.9	14.1 ± 3.0 13.6 ± 2.7	14.74 ± 3.06 14.71 ± 2.92	15.5 ± 2.6 15.4 ± 2.8

OMT: optimal medical therapy, CCM: cardiac contractility modulation, 6MWD: 6-minute walking distance, MLWHFQ: Minnesota Living with Heart Failure Questionnaire, SBP: systolic blood pressure, SD: standard deviation, LVEF: left ventricular ejection fraction, and VAT: ventilatory anaerobic thresholds.

**Table 2:** A summary of reported adverse events and frequency.

	*FIX-HF-5 Pilot [12]*		*FIX-CHF-4 [13]*		*FIX-HF-5 [20]*		*FIX-HF-5C [21]*	
	CCM (N =25)	OMT (N = 24)	CCM ON (N =160)	CCM OFF (N = 161)	CCM (N = 210)	OMT (N = 212)	CCM (N = 74)	OMT (N = 86)
General Cardiopulmonary Event	1	4	2	3	60	58	3	2

General Medical	3	10	1	6	98	81	7	7

Arrhythmia (VF, VT, AF, SVT)	1	2	6	4	40	30	3	2

Worsening HF	2	3	7	8	72	85	3	7

ICD/Pacemaker malfunction or Sensing Defect	1	0	1*∗*	0	13	7	2	0

Bleeding	NA	NA	NA	NA	8	8	0	1

Sepsis	NA	NA	NA	NA	11	2	1	1

Localized Infection	NA	NA	NA	NA	33	36	1	4

Neurologic Dysfunction	NA	NA	NA	NA	3	14	0	0

Thromboembolism (non-neurologic)	NA	NA	NA	NA	3	5	1	1

Optimizer Malfunction	NA	NA	1	NA	30	NA	6	NA

Total	8	18	20	22	371	326	27	25

Complications Related to CCM procedure/Device	2 lead dislodgements One event of “chest sensation” during CCM signal application resolved with parameter adjustment of signals 2 optimizer pocket infections 1 pericardial effusion 1 inappropriate ICD firing		3 Lead dislodgements 5 Device pocket infections 2 Pericardial effusion 4 Bleedings at CCM site		3 Lead fracture 6 RV Lead Dislodgement 6 RA lead dislodgement 3 CCM pocket dehiscence/erosion 2 CCM pocket infection 2 CCM pocket stimulation 1 CCM pocket bleeding 2 Lead perforation 2 Sensation due to CCM 1 Extracardiac stimulation		5 lead dislodgements 1 DVT 1 Generator Erosion requiring pocket revision and lead replacement	

CCM: cardiac contractility modulation, OMT: optimal medical therapy, VF: ventricular fibrillation, VT: ventricular tachycardia, AF: atrial fibrillation, SVT: supraventricular tachycardia, HF: heart failure, and DVT: deep vein thrombosis.

*∗* Due to either T-wave oversensing or the need for ICD lead repositioning.

## Data Availability

This is a meta-analysis of randomized controlled trials. All the data used are included within this manuscript.

## Additional Points

*Brief Summary.* Cardiac contractility modulation (CCM) is a novel therapy that targets patients with a limited device therapy options for symptomatic improvement and reducing heart failure admissions. More data are available now and with promising results. We hope to condense the studies into a concise systematic review and meta-analysis of the 4 major RCT. We would also like to focus on highlighting the efficacy, safety, and potential adverse effects of CCM in our study.

## Supplementary Materials

Supplementary Materials Supplemental Table 1: risk of bias of the individual studies by Cochrane Risk Assessment Tool.
