# Supplementary material for: Outcomes of Cardiac Contractility Modulation: A Systematic Review and Meta-Analysis of Randomized Clinical Trials
Source: Cardiovasc Ther. 2019 Jun 17;2019:9769724. doi: 10.1155/2019/9769724 (PMC6739758; doi:10.1155/2019/9769724)
Supplement: Supplementary Materials — Supplemental Table 1: risk of bias of the individual studies by Cochrane Risk Assessment Tool. [file 9769724.f1.docx]

**Supplemental Material**

**Supplemental Table 1: Risk of Bias of The Individual Studies by Cochrane Risk Assessment Tool**

|  | **FIX-HF-5 Pilot** | **FIX-CHF-4** | **FIX-HF-5** | **FIX-HF-5C** |
| --- | --- | --- | --- | --- |
| **Random sequence generation** *(Selection bias)* | 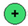 | 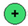 | 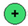 | 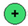 |
| **Allocation concealment** *(Selection bias)* | 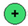 | 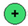 | 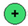 | 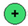 |
| **Blinding of participants and personnel** *(Performance bias)** | 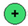 | 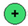 | 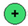 | 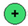 |
| **Blinding of outcome assessment** *(Detection bias)* | 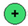 | 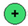 | 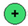 | 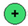 |
| **Incomplete outcome data** *(Attrition bias)* | 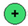 | 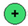 | 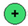 | 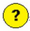* |
| **Selective reporting** *(Reporting bias)* | 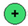 | 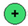 | 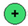 | 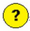* |
| **Other sources of bias** | 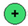 | 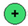 | 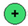 | 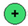 |


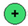
= Low risk of bias
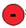
 = High Risk of bias
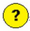
 = Unclear

* Through review of the manuscript and supplemental data did not yield sufficient individualized outcome data to allow for proper assessment and review of the reported outcomes
